# Are there biomechanical differences in repair technique for lesser tuberosity osteotomy with stemless total shoulder arthroplasty?

**DOI:** 10.1016/j.xrrt.2026.100794

**Published:** 2026-06-12

**Authors:** Emil Espinal, Mahdi Mazeh, Daniel Oravec, Johnny Kasto, Chimdindu Obinero, Kai Zhu, Yener N. Yeni, Shariff K. Bishai, Stephanie J. Muh, Jared M. Maylis

**Affiliations:** a Department of Orthopaedic Surgery, Henry Ford Health, Detroit, MI, USA; b Detroit Orthopaedic Institute, PLLC, Detroit, MI, USA

**Keywords:** Lesser tuberosity, Subscapularis repair, Total shoulder arthroplasty, Osteotomy, Bone tunnels, Suture anchor

## Abstract

**Background:**

Total shoulder arthroplasty (TSA) has evolved as a treatment for advanced shoulder osteoarthrosis, with stemless implants gaining popularity due to reduced risks of stress shielding and complications associated with stemmed designs. However, the optimal technique for repairing the subscapularis with lesser tuberosity osteotomy (LTO) during TSA remains under investigation. This study evaluates the biomechanical performance of two repair techniques—bone tunneling and suture anchors—used for LTO in the context of stemless TSA.

**Methods:**

Ten shoulders from 6 cadaveric specimens (3 males and 3 females; aged 58–93 years old) were prepared and allocated to bone tunneling or suture anchor repair groups. Cyclic loading tests were performed, followed by incremental load-to-failure protocols. Outcomes included repair gapping during cyclic loading, initial repair stiffness (derived as the average secant stiffness for 10 of the initial 3,000 cycles prior to fatigue), cycles to failure, displacement of repair, and load to failure.

**Results:**

The mean age of the cadavers used in this study was 90.2 ± 14.7 years. Mean failure load were alike, and cycles to failure nearly identical, between groups (*P* = .86-1.00). No significant differences were observed between the two groups in repair gapping during cyclic loading (*P* = .42). However, a nonsignificant trend was observed for initial repair stiffness (*P* = .07), with the tunnel group showing greater stiffness (62.5 ± 13.9 N/mm) compared to the anchor group (46.0 ± 10.6 N/mm). A trend was also noted in displacement of repair (*P* = .099), with the tunnel group exhibiting greater displacement (41.4 ± 17.3 mm) than the anchor group (23.0 ± 13.1 mm).

**Conclusion:**

This study suggests that both techniques may yield comparable biomechanical outcomes in cadaveric models of LTO repair, which warrants further exploration. As a pilot project, this study lays the groundwork for future research that could expand upon these preliminary results and contribute to a deeper understanding of LTO repair techniques.

Total shoulder arthroplasty (TSA) has become a mainstay in the treatment of primary shoulder osteoarthritis and other secondary degenerative shoulder diseases.^18^ Despite advancements since the inception of the total shoulder in the 1970s, there are a number of concerns that remain and drive the search for improved techniques and implants.^7^ In particular, stress shielding of the proximal humerus from a stemmed implant is an area of concern. A stemmed implant can introduce increased risk of complications such as loss of bone stock, intraoperative and postoperative periprosthetic fractures, and malpositioning of components.^3^ This has led to the development of the stemless shoulder arthroplasty. Several studies have shown that stemless TSA has equivalent patient outcomes, complications, and longevity to stemmed TSA.^1^^,^^4^^,^^6^^,^^10^^,^^15^^,^^16^ As the incidence of canal sparing implants has increased, 1 technical challenge that has lingered is repair of the subscapularis.^5^^,^^22^

The management of the subscapularis tendon during the surgical approach has seen variability between surgeons.^2^^,^^14^ There are 3 main techniques that surgeons use in their approach mobilizing the subscapularis tendon to gain access to the glenohumeral joint: subscapularis tenotomy, lesser tuberosity osteotomy (LTO), and subscapularis peel.^9^^,^^21^ There has not been a consensus on the most optimal approach to repair of the subscapularis with all 3 techniques showing good clinical outcomes.^19^ Okafor et al ^13^^,^^17^^,^^19^ found that surgeons, who performed stemmed TSA prior to use of stemless TSA, continued their preferred management of the subscapularis after converting to stemless TSA. Theoretical advantages of the LTO for TSA include improved mechanical strength and high rates of healing.^11^ The current evidence comparing the efficacy of the subscapularis peel vs. LTO in terms of cycles to failure, repair gapping, and load to failure have been limited to assessment of stemmed implants.^9^ Similarly, studies comparing LTO vs. subscapularis tenotomy have also shown greater healing and clinical outcomes with the LTO but again are limited to older stemmed implants.^8^ While more recent studies by Werner et al ^21^ have assessed the biomechanics of both subscapularis tenotomy and subscapularis peel with stemless TSA, there is paucity of research evaluating LTO repair techniques in stemless TSA.

The aim of this study was to elucidate the mechanical differences of the following two different LTO fixation techniques: bone tunneling vs. anchor-based repair in the setting of a stemless TSA. We hypothesize that significant differences will emerge between the two groups in terms of biomechanical function, providing valuable insights for optimizing subscapularis repair techniques in stemless TSA.

## Materials and methods

Ten cadaveric shoulders were acquired under institutional guidelines. This was an institutional review board exempt study as it did not involve human subjects (May 5, 2023, IRB no. 16376, Henry Ford Health). There were four bilateral pairs and two unilateral specimens from 6 human cadavers (3 males and 3 females; ages 58-93 years old). Demographic data for each specimen was recorded including sex, body mass index, history of tobacco use, medical comorbidities, and cause of death (Table I). Exclusion criteria were specimens <18 years of age and evidence of prior rotator cuff surgery. Specimens were stored at −20 °C until the day of planned specimen preparation and testing. Specimens were left to thaw for approximately 12 hours before preparation ensued.

**Table I:** Specimen demographic data.

Specimen	Age, years	Sex	BMI, kg/m^2^	Comorbidities	Tobacco use (ppd, years)	Cause of death
SH001	86	F	16.5	Multiple sclerosis	Unknown	Pneumonia
SH002	58	M	28.2	COPD and TIIDM	1-2 ppd, 10+ yr	Cardiac dysrhythmia
SH003	86	F	15.9	Asthma	Unknown	Encephalopathy
SH004	-	-	-	-	-	-
SH005	93	F	23.6	Coronary artery disease	Unknown	Myocardial infarction
SH006	-	-	-	-	-	-
SH007	91	M	16.3	Bladder cancer and heart disease	1-2 ppd, 10+ yr	Bladder cancer
SH008	-	-	-	-	-	-
SH009	87	M	23.4	Bladder cancer and prostate cancer	Unknown	Bladder cancer
SH010	-	-	-	-	-	-

*BMI*, body mass index; *COPD*, chronic obstructive pulmonary disease; *F*, female; *M*, male; *ppd*, pack per day, *TIIDM*, type II diabetes mellitus.

Dashes (-) indicate that demographic data are identical to the previous listed specimen, as multiple specimens were obtained from the same donor.

For each specimen, the overlying skin, deltoid, and pectoralis major muscles were removed and the rotator cuff of each specimen was inspected. Any damage to the rotator cuff was noted. The entire rotator cuff musculature was then elevated from the scapula using sharp dissection and the scapula removed. The rotator interval was opened and the long head of the biceps tenotomized at the superior glenoid insertion. For all specimens, a LTO was performed starting laterally at the biceps groove. Creation of the LTO was performed using previously described techniques.^6^ Osteotomy was started with a thin 20-mm wide oscillating saw with the goal to create a thin osteotomy size 3-4 mm thick and an osteotome was used to complete the cut. After completion of the LTO, an inferior, medial, and posterior capsular release was performed sharply, and the humeral head was exposed circumferentially. The humeral head was osteotomized with an anatomic neck cut using the oscillating saw. The cut was made matching the patient's native version and inclination.

Five specimens were allocated to each of the following two types of LTO repair groups: a bone tunnel transosseous repair of LTO (“Tunnel” group) and knotless anchor-based repair of LTO (“Anchor” group). For the transosseous bone tunnel technique, two bone tunnels were drilled starting in the bicipital groove and out of the humeral head cut surface. Two strands of No. 2 FiberWire (Arthrex, Naples, FL, USA) were looped on each other, and the free ends were passed through the drill holes. Using a free needle, these free ends were passed through the undersurface of the subscapularis tendon (deep to superficial) so that the looped end of suture lay lateral to the LTO and the free ends lay medial. The suture was tensioned and tied over the LTO in Nice knot fashion and secured with alternating half hitches. For the anchor repair, two all-suture anchors (Iconix, Stryker, Portage, MI, USA) loaded with 1.4-mm nonabsorbable suture were placed at the subscapularis footprint medial to the lesser tuberosity. The 4 free ends of the suture were then passed through the subscapularis tendon medial to the LTO. One suture end from each anchor was fed into a knotless 3.9-mm (Omega, Stryker, Portage, MI, USA) lateral row anchor and appropriately tensioned, ensuring reduction of the LTO. The anchors were placed in the bicipital groove, creating a construct of two medial and two lateral row anchors.

The humerus was then sized for an appropriate stemless humeral arthroplasty (Easytech, FX Shoulder, Dallas, TX, USA). The humeral component anchor bases were impacted into the cut humeral surface. Prepared specimens were cut at the humeral shaft 14.5 cm distal to the repair site using the oscillating saw.

For each specimen, the humeral shaft was potted using automotive body filler (3M Bondo, St. Paul, MN, USA) within a 10-cm length of Polyvinyl Chloride pipe (leaving approximately 4.5 cm from surface of filler to the center of the repair). To ensure consistent alignment during potting, a jig was utilized with a post fit inside the medullary canal. The potting material was allowed to cure for 2 hours and specimens were stored at 5 °C until testing. Throughout potting, exposed portions of the specimen were wrapped in saline-soaked towels wrapped in plastic wrap.

The subscapularis muscle was constrained in a cryogenic clamp with a serrated gripping surface at 90° relative to the humeral shaft, with the Polyvinyl Chloride pipe constrained by 6 screws in an aluminum fixture. The distance between the repair and cryogenic grip was fixed at 6.5 cm for all specimens. Specimens were kept moist throughout testing by spraying 37 °C saline, and dry ice in cryogenic clamps was replenished throughout the test in 10-minute intervals.

**Figure 1:**
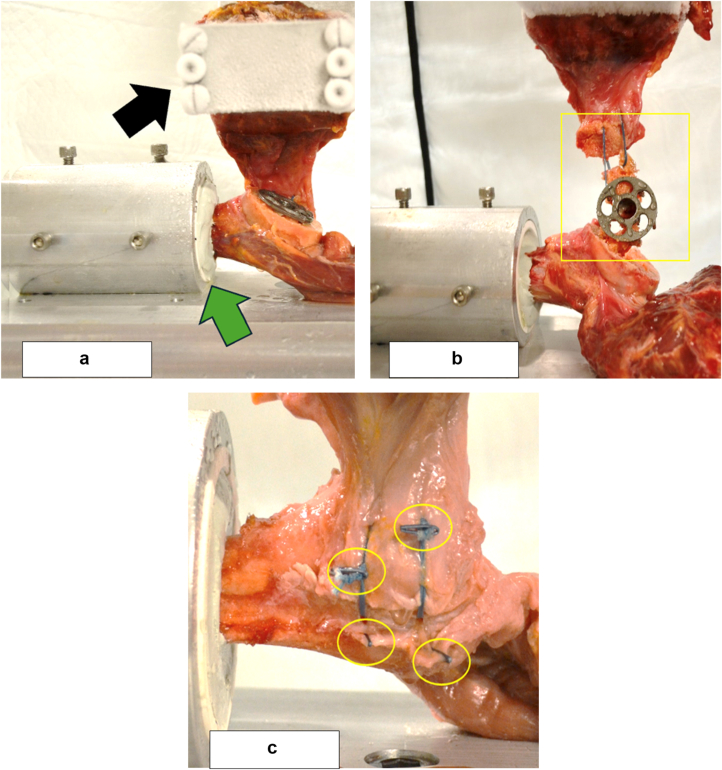
Experimental setup and representative failure of subscapularis repair constructs. (**a**) Potted humerus (*green arrow*) with the subscapularis tendon secured in a cryogenic clamp (*black arrow*). (**b**) Representative repair failure following cyclic loading with an incrementally increasing load protocol (*yellow square*). (**c**) Vascular clip placement used for displacement tracking in a bone tunnel repair specimen (*yellow circles*).

In order to assess repair gapping occurring due to cyclic loading, vascular clips (Ethicon Ligaclips, Cincinnati, OH, USA) were placed medial and lateral to the LTO prior to mechanical testing. Figure 1 illustrates the experimental assembly. It demonstrates the cryogenic clamp and potted humerus (1A), a representative image of repair failure (1B), and an example of vascular clip placement for repair gap measurement (1C).

**Figure 2:**
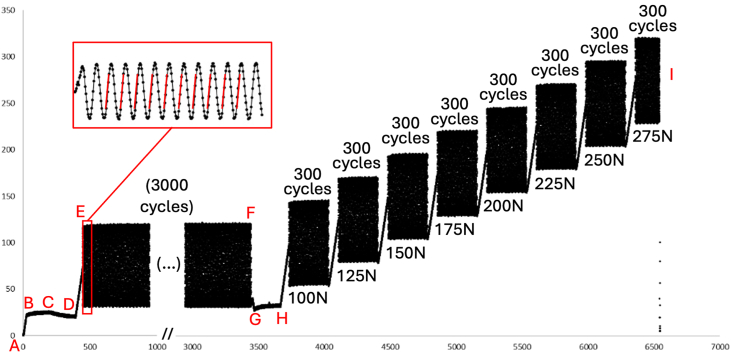
Representative plot of loading scheme. Specimens were loaded from initial contact to 25 N (A-B) and held for 3 minutes to eliminate tendon crimp (B-C). Repair gapping was then recorded (C-D). Specimens subsequently underwent 3,000 cycles of sinusoidal loading at 75 ± 50 N (E-F), after which repair gapping was recorded again (G-H). Incrementally increasing cyclic loading was then applied until repair failure (H-I).

Specimens were loaded from contact to 25 N at 1 N/s (A to B, Fig. 2), and 25 N was held under force control for 3 minutes to eliminate crimp.^6^ After the 25 N hold period, the clip-to-clip distance (center of clip to center of clip) was measured between pairs of distal and proximal clips (C to D, Fig. 2) using a digital caliper, and the average between the individual distal and proximal distances was recorded (CC_1_). Specimens were then loaded to 75 N at 1 N/s (D to E, Fig. 2) and subjected to 3,000 cycles of cyclic loading (75 ± 50 N at 1 Hz) (E to F, Fig. 2). For specimens that survived the initial 3,000 cycles, a second set of clip-to-clip distances (CC_2_) were measured (G to H, Fig. 2). Finally, specimens were subjected to bouts of cyclic loading, with a constant amplitude (±50 N) and frequency (1 Hz) but a central load incrementally increasing in 25 N from 100 N to 450 N or until failure. The specimens were subjected to 300 cycles at each central load level (H to I, Fig. 2).

Repair gapping due to cyclic loading was calculated as the difference in mean distance before and after the initial 3,000 cycles for those that survived the initial bout of cyclical loading (CC_2_-CC_1_; measurements made at time points D and G, Fig. 2). Initial stiffness was derived as the average secant stiffness for 10 of the initial 3,000 cycles. Failure load was recorded as the central load of the cycle at which failure occurred (ie, in example Fig. 2, 275 N for failure cycle at time point I). The total number of cycles to failure (between E and I, Fig. 2) was recorded and expressed in terms of 75 N-equivalent cycles using the Palmgren-Miner linear cumulative damage model.^6^ Finally, extension to failure during cyclic loading was derived as the difference in actuator displacement occurring between the first cycle and the last cycle prior to failure (displacement from time points E to I, Fig. 2).

Student *t*-test was used to assess the differences in measured outcomes between two specimen groups (tunnel and anchor) with significance set as *P* = .05. For nonsignificant variables, the sample size per group to demonstrate differences at *P* = .05 with statistical power of 0.80 was estimated.

## Results

Two specimens from each group did not survive the initial 3,000 cycles. For the two failed tunnel specimens, failure occurred at 652 and 2,771 cycles, and for the two failed anchor specimens, failure occurred at 65 and 897 cycles. For specimens that did not survive the initial 3,000 cycles, repair gapping occurring during cyclic loading was therefore incalculable and not included in statistical analyses.

**Figure 3:**
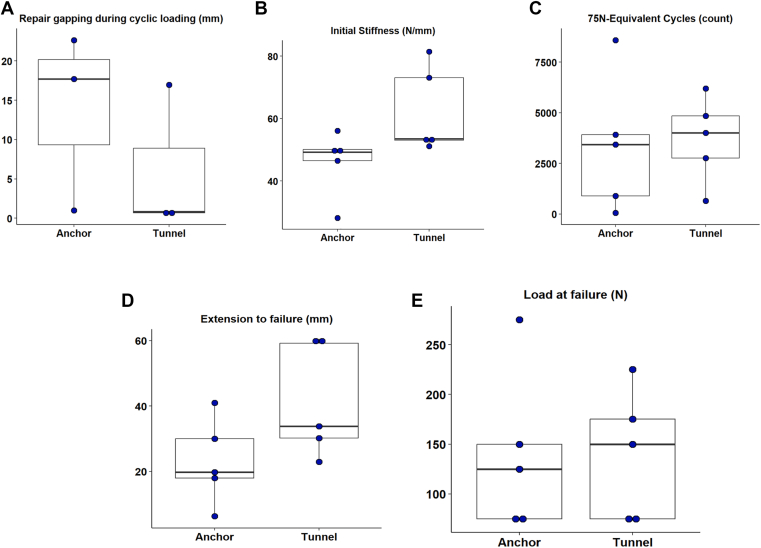
Comparison of biomechanical outcomes between anchor and bone tunnel repair constructs. (**A**) Repair gapping during cyclic loading, measured in millimeters. (**B**) Initial construct stiffness, expressed in N/mm. (**C**) Number of cycles completed at a 75-N–equivalent load prior to failure. (**D**) Extension to failure, measured in millimeters. (**E**) Load at failure, measured in Newtons.

None of the measured outcomes were statistically different between tunnel and anchor specimen groups (Table II). Mean failure load level was identical and mean 75 N-equivalent cycles were nearly identical between the two groups. There was a nonsignificant trend toward higher extension to failure (*P* < .1) and stiffness (*P* < .08) in the tunnel group (Fig. 3). Based on sample size calculations, differences in extension to failure and stiffness may be demonstrable with a larger sample size (11 and 9 per group, respectively).

**Table II:** Results from statistical analyses comparing tunnel and anchor groups.

Variable	Tunnel (Av ± SD)	Anchor (Av ± SD)	*P* value∗
Repair gapping during cyclic loading (mm)	6.1 ± 9.4	13.8 ± 11.3	.42
Initial stiffness (N/mm)	62.5 ± 13.9	46.0 ± 10.6	.07
75N-Equivalent cycles to failures	3,694 ± 2,107	3,376 ± 3,334	.86
Extension to failure (mm)	41.4 ± 17.3	23.0 ± 13.1	.10
Load at failure (N)	140 ± 65	140 ± 82	1.00

*Av*, average; *SD*, standard deviation.

∗ *P* value from *t*-test.

## Discussion

There has been a lack of consensus regarding the optimal subscapularis exposure and repair technique in the context of TSA. While some studies have shown no biomechanical difference in load failure between LTO and subscapularis tenotomy, much of the evidence points to equivocal outcomes with different subscapularis exposure techniques. However, other studies demonstrate superior strength with LTO. Ponce et al^19^ found significantly higher strength after cyclic loading and higher load failures in LTO compared to tenotomy. Krishnan et al^13^ found similar results in cadaveric models and also found good clinical results with LTO and double row repair. Another potential advantage of the LTO is the ability to perform a subscapularis tenotomy in the revision setting, without having to expose through a previously repaired tendon. With evidence supporting the use of LTO, we focused our study on this technique. This study aimed to compare the biomechanical performance of two different LTO repair techniques, bone tunneling and suture anchors, within the context of stemless TSA. Our findings indicate that both techniques yield comparable biomechanical outcomes in a cadaveric model, though some trends were noted in stiffness and displacement. However, no statistically significant differences were found in key variables such as repair gapping, cycles to failure, or load at failure. This suggests that while both techniques may be viable for LTO repair in stemless TSA, further studies with larger sample sizes are needed to determine the superiority of either approach definitively.

One of the key findings in this study was the trend toward higher initial repair stiffness in the bone tunneling group compared to the anchor group (*P* = .07). This suggests that bone tunneling may provide a more rigid repair construct initially, which could be beneficial in resisting early displacement forces. However, despite this trend, no statistically significant differences in repair gapping during cyclic loading or ultimate load to failure were observed between the two groups. Additionally, our results indicated a trend toward greater displacement in the bone tunneling group (41.4 ± 17.3 mm) compared to the anchor group (23.0 ± 13.1 mm, *P* = .099); displacements that would both be considered clinical failures. This could suggest that although bone tunneling initially provides a stiffer construct, it may not necessarily translate to a reduced gap formation under cyclic loading.

The initial postoperative period is arguably the most important with respect to repair success as the tendon load is solely dependent on the repair fixation.^20^ Increased gap formation may translate to decreased osteotomy union rates, though this has not been confirmed in the literature. In their study of anatomic TSA performed with LTO, Johnson et al^11^ reported excellent union rates. If increased gapping at the repair site leads to decreased union rates, further research is needed to determine the clinical relevance of union. Levy et al^15^ performed a retrospective review of TSA patients with LTO radiographic union, nondisplaced nonunion, and displaced nonunion. They found that the displaced nonunion group had lower PROMs than the union and nondisplaced nonunion group, but all groups had increased PROMs and range of motion compared to preoperative measures. They also found high satisfaction in all groups. Further research is needed to determine whether this increased displacement in the bone tunnel group affects long-term functional outcomes.

Our study's findings are in line with previous research comparing various LTO repair techniques. For instance, Krishnan et al^13^ found high biomechanical strength in both single-row and double-row transosseous LTO repair. However, suture anchor repairs may offer advantages in surgical efficiency and reproducibility, potentially making them a more favorable option in clinical settings.

The question of optimal LTO fixation remains debated in the literature. While no direct comparison of bone tunnel vs. suture anchor fixation has been studied in regard to LTO, there have been cadaveric studies demonstrating superior strength with bone tunnels vs. suture anchors for Bankart repair.^12^ Our study adds to this growing body of evidence by demonstrating that while both techniques are viable, neither approach demonstrated clear biomechanical superiority in our cadaveric model.

## Limitations

The study has limitations. First, all of the specimens were not paired. As a result of our specimen collection, two of the shoulders were from different cadavers. This could lead to a sampling bias as differences in the cadavers could not be accounted for when comparing the two LTO repair techniques.

Second, demographics of the specimens may have affected the repair techniques and subsequent testing. As with many biomechanical cadaveric studies, the demographic and biologic state of the specimen is not representative of the typical patient population who would receive surgical intervention by surgeon preference. For example, one of the specimens was from an individual who smoked 1-2 packs of cigarettes a day and had type II diabetes. Many surgeons ensure a patient's diabetes is well controlled before offering surgical intervention. Additionally, smoking is a prohibitive factor for many surgeons, particularly in arthroplasty. Another specimen was from a 91-year-old with active bladder cancer and heart disease; a patient who would be an unlikely surgical candidate. Additionally, the study was limited by the variability in cadaveric specimen quality, particularly given the advanced age of the donors (mean age 90.17 years). Age-related changes in bone density and tendon quality could have influenced the biomechanical properties observed in this study. Future studies should explore the impact of bone quality on LTO repair outcomes, as well as investigate patient-specific factors such as tendon integrity and muscle atrophy.

As a pilot study, our sample size was limited to 10 specimens, reducing the statistical power to detect small but potentially meaningful differences between repair techniques. Based on our sample size calculations, a larger study with at least 11 specimens per group may be necessary to confirm trends in extension to failure and stiffness. Furthermore, our use of cadaveric specimens introduces inherent limitations, as cadaveric tissue does not fully replicate the biological healing process that occurs in vivo. Future studies should consider in vivo models or clinical trials to better assess the long-term implications of these repair techniques.

## Conclusion

This study provides preliminary evidence that both bone tunneling and suture anchor techniques are viable options for LTO repair in the context of stemless TSA. While no statistically significant differences were observed between the two techniques, trends suggest that bone tunneling may offer greater initial stiffness, whereas anchor-based repairs may reduce displacement. Given the clinical significance of subscapularis healing and function in TSA outcomes, further research with larger sample sizes and clinical follow-up is warranted to optimize LTO repair techniques. Our findings underscore the need for continued exploration of biomechanical and clinical outcomes to guide surgical decision-making in TSA.

## Disclaimers:

Funding: This study was supported by the ASES Foundation (FX Shoulder Program) and Stryker (Funding ID Number: F20531). The funders had no role in the study design, data collection, analysis, interpretation of results, manuscript preparation, or the decision to submit the article for publication.

Conflicts of Interest: Shariff K. Bishai or an immediate family member has professional activities with Johnson and Johnson, Shoulder Innovations, Inc., Atreon, Pacira Pharmaceuticals Incorporated, Smith and Nephew, CONMED Corporation, and BARD; holds stock in Atreon; intellectual property with Smith and Nephew; and serves on the editorial or governing board of Journal of Shoulder and Elbow Surgeons and Journal of Orthopedic Experience and Innovations, serves as the Chair of the Orthopedic Video Theater for the American Academy of Orthopedic Surgeons.

Stephanie J. Muh or an immediate family member has professional activities with Enovis, American Orthopedic Association, Journal of Shoulder and Elbow Surgery International, Smith and Nephew, Exactech, Inc., and American Academy of Orthopedic Surgeons; serves on the Board of Directors or as a Committee member of the American Shoulder and Elbow Surgeons (ASES); and serves on the editorial or governing board of the Journal of Shoulder and Elbow Surgery International.

Jared M. Mahylis or an immediate family member has professional activities with Exactech, Inc.; professional activities, intellectual property, and IP royalties with FX Shoulder USA, Inc; and serves as a Board of Directors or Committee member of the American Academy of Orthopedic Surgeons (AAOS).

Any additional authors, their immediate family, and any research foundation with which they are affiliated have not received any financial payments or other benefits from any commercial entity related to the subject of this article.
